# Histone Deacetylase Inhibitors Promote the Anticancer Activity of Cisplatin: Mechanisms and Potential

**DOI:** 10.3390/ph18040563

**Published:** 2025-04-11

**Authors:** Yang Zhou, Qun Luo, Liangzhen Gu, Xiao Tian, Yao Zhao, Yanyan Zhang, Fuyi Wang

**Affiliations:** 1 Beijing National Laboratory for Molecular Sciences, CAS Research/Education Center for Excellence in Molecular Sciences, CAS Key Laboratory of Analytical Chemistry for Living Biosystems, Institute of Chemistry, Chinese Academy of Sciences, Beijing 100190, Chinaqunluo@iccas.ac.cn (Q.L.);; 2 University of Chinese Academy of Sciences, Beijing 100049, China; 3 College of Traditional Chinese Medicine, Shandong University of Traditional Chinese Medicine, Jinan 250355, China; 4 National Centre for Mass Spectrometry in Beijing, Beijing 100190, China

**Keywords:** cisplatin, HDAC inhibitor, histone acetylation, DNA lesion, activity enhancement

## Abstract

Cisplatin is a widely used DNA-targeting anticancer drug. Histone deacetylase inhibitors (HDACi) cause histone hyperacetylation, changing chromatin structure and accessibility of genomic DNA by the genotoxic drug. As a consequence, HDACi could promote cisplatin cytotoxicity. Hence, the underlying mechanisms by which HDACi alter the action pathways of cisplatin to promote its anticancer activity have attracted increasing attention during the past decades. It has been commonly accepted that HDACi elevate the acetylation level of histones to release genomic DNA to cisplatin attack, increasing the level of cisplatin-induced DNA lesions to promote cisplatin cytotoxicity. However, how the HDACi-enhanced cisplatin lesion on DNA impacts the downstream biological processes, and whether the promotion of HDACi to cisplatin activity is attributed to their inherent anticancer activity or to their induced elevation of histone acetylation, have been in debate. Several studies showed that HDACi-enhanced DNA lesion could promote cisplatin-induced apoptosis, cell cycle arrest, and reactive oxygen species (ROS) generation, subsequently promoting cisplatin efficiency. In contrast, HDACi-induced elimination of ROS and inhibition of ferroptosis were thought to be the main ways by which HDACi protect kidneys from acute injury caused by cisplatin. Based on our recent research, we herein review and discuss the advances in research on the mechanisms of HDACi-induced enhancement in cisplatin cytotoxicity. Given that histone acetyltransferase (HAT) inhibitors also show an effect enhancing cisplatin cytotoxicity, we will discuss the diverse roles of histone acetylation in cancer therapy in addition to the synergistic anticancer effect and potential of HDACi with genotoxic drugs and radiotherapy.

## 1. Introduction

Cisplatin (*cis*-diamminedichloroplatinum(II), cDDP, or DDP), which was first synthesized and reported by Italian chemist Michele Peyrone, is a small and simple complex composed of one platinum atom coordinating to two ammines and two chlorides [1]. In 1965, Rosenberg occasionally discovered its antiproliferation effect on *E. coli* [2] and further verified its anticancer function [3]. In 1978, cisplatin was approved by the Food and Drug Administration (FDA) of the US for the treatment of solid tumors [4]. During the decades, cisplatin and the second and third generations of platinum drugs, carboplatin and oxaliplatin, have been the first-line chemotherapeutics and are currently involved in more than half of the treatment regimens for cancer patients in clinics [5].

The primary biological target of cisplatin is thought to be genomic DNA [6]. Cisplatin modification on DNA produces distinct changes in the structure of DNA duplexes, causing DNA lesions and stimulating intercellular responses, including cell cycle arrest and DNA repair [6,7]. This subsequently induces apoptosis and necrosis in cancer cells when cells fail to repair cisplatin-induced DNA damage [8,9]. In addition, cisplatin has been shown to be a reactive oxygen species (ROS) producer, contributing partially to cisplatin cytotoxicity [10]. However, the application of cisplatin in clinics is limited by severe side effects and tissue toxicity, such as nephrotoxicity, ototoxicity, and neurotoxicity [11,12]. Moreover, drug resistance, including intrinsic resistance and acquired resistance, also reduces cisplatin efficacy, hampering the clinical application of the drug [8]. This stimulates a significant number of studies to explore how to promote the effectiveness of cisplatin while reducing its side effects and drug resistance.

It has been reported that the histone deacetylase (HDAC) inhibitors, such as trichostatin A (TSA) and suberoylanilide hydroxamic acid (SAHA), could promote the cytotoxicity of cisplatin [13,14] by elevating the level of histone acetylation [15,16]. These findings have inspired extensive studies on the underlying mechanisms by which HDAC inhibitors enhance cisplatin cytotoxicity so as to maximize the drug’s effectiveness while minimizing its side effects. For instance, how the acetylation of histones impacts the action of cisplatin to lead to higher cytotoxicity towards cancer cells, and the acetylation of which histone(s) and the acetylation of which residues in the histones most contributes to the promotion of cisplatin efficacy. In this review, based on a recent study of ours [17], we aim at providing a detailed description and evaluation of HDAC inhibitors, the correlations between cisplatin efficacy and histone acetylation, and the potential of HDAC inhibitors for promoting the clinical application of cisplatin. We will first briefly introduce the classification and biological functions of HDAC and HDAC inhibitors and then review and discuss the advances in research in the molecular mechanisms by which HDACi promote cisplatin anticancer activity and reduce the toxic side effects of the drug. We will also look at the potential synergistic effect of the combination of HDACi with cisplatin and other DNA-targeting anticancer drugs in the clinic. We anticipate that the present information could facilitate further research and promotion of the clinical effectiveness of first-line anticancer drugs.

## 2. Histones and Histone Acetylation

**Figure 1:**
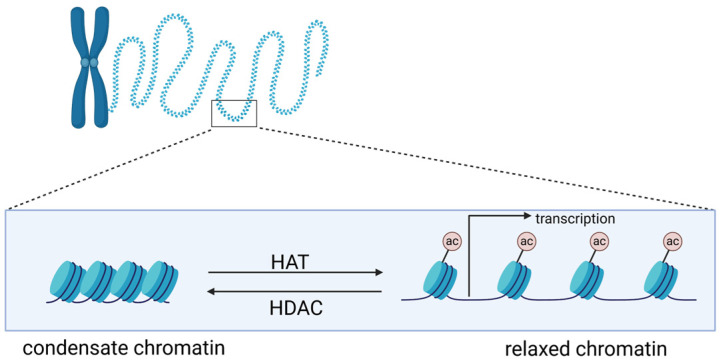
Impact of histone acetylation on chromatin structure and downstream biological processes such as transcription.

Histones are a family of proteins that provide structural support for chromatin, which further condenses to form chromosomes. In eukaryotes, DNA in chromatin is organized in arrays of nucleosomes [18], and two copies of each histone protein (H2A, H2B, H3, and H4) are assembled into an octamer around which 146 base pairs of DNA wraps to form a nucleosome core [19]. Nucleosomes play a crucial role as the principal packaging element of genomic DNA, of which the structures are determinant for DNA accessibility by endogenous and exogenous molecules, including drugs [20]. Histones have a flexible and charged NH_2_-terminus (also termed histone tail) that protrudes from the nucleosome, and their posttranslational modifications, like methylation and acetylation, could alter chromatin architecture [21]. The level of histone acetylation is determined by the activities of histone acetyltransferases (HATs) and histone deacetylases (HDACs), which reversibly regulate histone acetylation [22]. HATs catalyze the transfer of acetyl groups to lysine residues, which could neutralize the positive charge of lysine, such that the interaction of histones with negatively charged DNA would be weakened, resulting in an increase in DNA accessibility and transcription activation. In contrast, HDACs remove acetyl groups from histones, leading to chromatin condensation [23,24]. Thus, inhibiting HDAC activity could increase the level of acetylation of histones, relaxing genomic DNA in chromatin to proteins and genotoxic drugs [20]. The former could activate specific transcription (Figure 1), and the latter may promote cytotoxicity of genotoxic drugs like cisplatin.

Mammalian cells encode 11 classic histone deacetylases, namely HDAC1, HDAC2, …, and HDAC11. These enzymes have different structures, functions, and subcellular localizations and are categorized into 4 groups, i.e., class I, IIa, IIb, and IV [25]. Class I HDACs, including HDAC1, 2, 3, and 8, and class IV HDAC (HDAC11) are located in the nucleus, while class II HDACs are found in both the nucleus and cytoplasm [26]. HDACs utilize zinc for catalytic sites and share histones as substrates [27]. Meanwhile, HDAC6, as a main cytoplasmic class IIb HDAC, could deacetylate non-histone proteins like HSP90 and α-tubulin [28]. Another class IIb HDAC, HDAC10, also shows the function of deacetylating HSP90 and α-tubulin [26,29]. HDAC11 predominantly resides in the cell nucleus. However, a coimmunoprecipitation study showed that it might be involved in the complex containing HDAC6, thus presenting in cytoplasm [30]. HDAC11 could also reduce α-tubulin acetylation to promote the meiotic process [31,32]. Studies also showed that p53 and NF-κB are HDAC substrates [33,34].

Aberrant expressions of HDACs occur in many types of cancers. For example, HDAC1 is upregulated in breast, prostate, gastric, and colon cancers [35,36,37,38]; HDAC2 is found to be overexpressed in cervical, gastric, and colorectal tumors; and HDAC3 has a high expression level in colon carcinomas [38]. HDACs play an important role in chromatin relaxation and gene transcription [39,40], DNA repair [41], and cytoskeletal recombination and cell migration [42]. Thus, HDACs regulate multiple cancer-relevant biological processes, showing different functions in different types of cancer cells (Table 1) [43]. HDAC1, 2, and 3 impact the transcription of genes associated with proliferation and apoptosis of cancer cells, increasing cancer cell proliferation and preventing apoptosis [44,45,46,47]. HDAC1 also controls the cell cycle via changing expression of CDK inhibitors, p21 and p27 [48]. Moreover, HDAC1 is involved in multidrug resistance in neuroblastoma cell lines [49], and HDAC4/6 elevate HIF-α expression, activating transcription of genes associated with tumor angiogenesis [50]. In addition, HDAC6 is involved in TGF-β/SMAD3-induced epithelial–mesenchymal transition (EMT) through deacetylating α-tubulin [51]. The α-tubulin deacetylation catalyzed by HDAC6 could also promote migration of cancer cells [52]. HDAC5 represses the activity of transcription factor GATA-1, and its nucleocytoplasmic distribution is associated with murine erythroleukemia (MEL) cell differentiation [53]. HDAC7 influences endothelial cell morphology, migration, and capacity to form capillary tube-like structures but does not alter cell adhesion, proliferation, or apoptosis [54]. HDAC8 alters cancer cell proliferation, cell cycle, and differentiation [55], and HDAC9 could mediate cancer cell proliferation, autophagic flux, and chemoresistance [56]. Thus, high HDAC9 expression confers tumors with invasive and angiogenic potential and poor prognosis [57,58]. HDAC10 could inhibit ferroptosis through reducing SLC7A11 expression and GSH levels, which elevates ROS levels and Fe^2+^ content. As a result, HDAC10 promotes proliferation and is also associated with poor prognosis of cancers [59,60]. Meanwhile, HDAC10 might be involved in DNA damage repair [60,61]. Similarly, HDAC11 promotes cancer proliferation and suppresses apoptosis [62], in addition to increasing Sox2 expression, maintaining cancer stem cell self-renewal, and cisplatin resistance [63].

Sirtuins (SIRTs) act as a group of NAD^+^-dependent deacetylases [111] and are also involved in genomic stability and DNA repair, cell metabolism and stress resistance, and cell fate and organism lifespan through altering acetylation levels of proteins, in particular histones. SIRTs regulate many biological processes and play diverse roles in cancer progression. For instance, SIRT2/6/3/4 function as tumor suppressors and are downregulated in cancer cells [94,98,99,112]. SIRT2 deacetylates H4K16, which might be conducive to the formation of condensed chromatin in mitosis and the remodeling of chromatin structure when cells exit from the cell cycle [113]. Moreover, SIRT2 also deacetylates APC/C and CDC20 to regulate mitosis and genome integrity [94], and SIRT6 protein acts as H3K9 and H3K56 deacetylase, changing gene expression, modulating telomeric chromatin, regulating DNA repair, and promoting genomic stability [114,115,116,117,118,119]. Moreover, SIRT6 is a tumor suppressor and acts to suppress cancer glycolysis and glutamine metabolism [112]. Mitochondrial sirtuins, SIRT3 and SIRT4, regulate mitochondrial proteins. While SIRT3 reduces stress-induced superoxide levels and maintains genomic stability to regulate mitochondrial physiology and to decrease tumorigenic potential [98], SIRT4 represses mitochondrial glutamine metabolism, inhibiting glutamine-dependent proliferation [120]. Given that glutamine metabolism block is required for DNA damage responses, SIRT4 plays a critical role in DNA damage response pathways and genomic stability [99]. However, another mitochondrial sirtuin, SIRT5, plays an oncogenic role via facilitating cancer cell growth and drug resistance [102]. SIRT7 deacetylates H3K18ac, which stabilizes the transformed state of cancer cells and is associated with tumor aggressiveness and poor prognosis [109]. Moreover, it has been shown that SIRT1 might also be an oncogene protein, which deacetylates and represses the activity of p53, attenuating p53-mediated apoptosis under conditions of DNA damage or oxidative stress [86,87]. Furthermore, SIRT1 interacts with AKT and ERRβ to promote breast cancer formation and development [88,89]. NF-κB was also shown to be a direct target of SIRT1 and might promote cell migration and invasion through MMP-2/9 [90]. Nevertheless, SIRT1 was shown to have tumor suppressor function. It deacetylates and represses expression of E2F1, affecting cellular sensitivity to DNA damage [91]. When DNA damage occurs, SIRT1 could re-localize from gene targets to DNA break sites to stimulate DNA repair [92]. When SIRT1 reduces acetylation of H3K9 to inhibit the expression of an antiapoptotic protein, survivin, it can inhibit tumor formation and growth [93].

## 3. Histone Deacetylase Inhibitors

**Figure 2:**
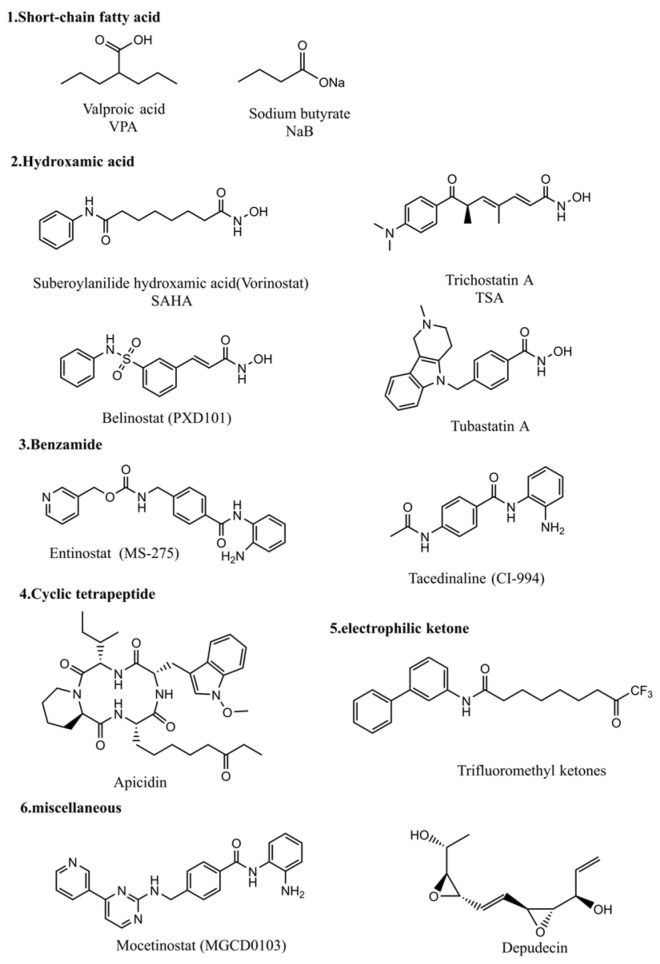
Chemical structure of HDAC inhibitors reported in the literature.

Given that histone acetylation level alters the structure of chromatin and the accessibility of genomic DNA by endo/exogenous biological molecules, impacting many intrinsic biological processes and pathological processes, in particular cancer cell growth and apoptosis [43], many small molecular HDAC inhibitors (HDACi) have been synthesized and used to explore the crucial roles of histone acetylation in genomic stability and transcription processes [121,122]. Meanwhile, the anticancer potential of HDACi has also attracted a lot of attention [122,123]. The inhibitors of zinc-dependent HDACs often contain zinc-chelating groups that disrupt enzyme–metal coordination at the active site, thus blocking catalytic activity of HDACs [27]. This type of HDAC inhibitor is generally composed of three structural elements, i.e., a cap, a functional (metal-binding) group, and a linker [123]. Based on their structural characters, HDAC inhibitors could be classified as short-chain fatty acids, hydroxamates, benzamides, cyclic tetrapeptides, electrophilic ketones, and miscellaneous (Figure 2).

**Figure 3:**
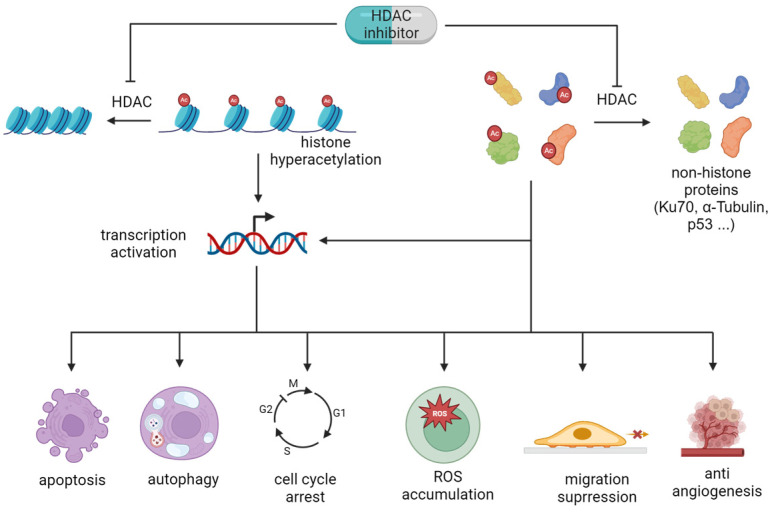
Biological functions of HDACi via altering the acetylation level of histone/non-histone proteins.

HDAC inhibitors elevate the acetylation level of histones and/or non-histone proteins, altering gene transcription and expression as knocking down/out of HATs does [124,125,126], consequently changing cell phenotypes, e.g., apoptosis, cell cycle arrest, and autophagy (Figure 3). For example, HDAC inhibitors, TSA, VPA, Apicidin, MS275, and SAHA (Figure 2) promote the expression of proteins in the death receptor pathway, such as TRAIL, DR5, Fas, and FasL, inducing apoptosis of leukemia cells [127,128,129]. VPA and romidepsin could lead to histone hyperacetylation, increasing CD20 expression and conquering rituximab resistance in B-cell lymphomas [130]. With these virtues, a number of HDAC inhibitors have shown attractive anticancer potential via inducing cancer cell apoptosis [131] and/or cell cycle arrest [132], generating reactive oxygen species (ROS) [133], suppressing angiogenesis and cell invasion [134,135], or increasing immunity response [136].

TSA is a well-known HDAC inhibitor. It increased the expression of Fas/FasL, which in turn triggered the activation of caspase-8 and activated the death-receptor apoptosis pathway [137]. TSA could also trigger mitochondrial membrane depolarization, releasing cytochrome c and activating caspase-3 to initialize the intrinsic apoptosis pathway [138]. This HDAC inhibitor also upregulated pro-apoptotic Bim and downregulated the anti-apoptotic Mcl-1 [138], while it promoted ROS production [138,139] and induced autophagy [138,140]. In U2OS cells, TSA promoted autophagy by inhibiting the mTOR (mammalian target of rapamycin) signaling pathway and enhancing forkhead box O1 (FOXO1) transcriptional activity [141], while it induced autophagy through regulating the PRMT5/STC1/TRPV6/JNK pathway in cervical cancer cells [142]. Moreover, it has been reported that TSA could promote the acetylation level of Ku70, a Bax-binding protein, leading to Bax release and activation, in turn triggering cell death [143]. TSA can also promote HSP90 acetylation, which causes release and degradation of the HSP90 client proteins RASGRP1 and CRAF, leading to downregulation of the mitogen-activated protein kinase (MAPK) signaling pathway and upregulation of the proapoptotic factor BIM [144]. On the other hand, TSA suppressed HIF-1α protein expression and VEGF transcription to target tumor angiogenesis, probably in a proteasome-dependent manner [145,146], while it arrested the cell cycle at the G2/M phase via reducing the expression of cyclin B and stimulating the expression of p21, an inhibitor of CDK1 [132]. In A549 cells, TSA was found to reverse TGF-β1-induced snail and slug expression, inhibiting migratory ability and TGF-β1-induced EMT of the cells (Figure 3) [147].

**Figure 4:**
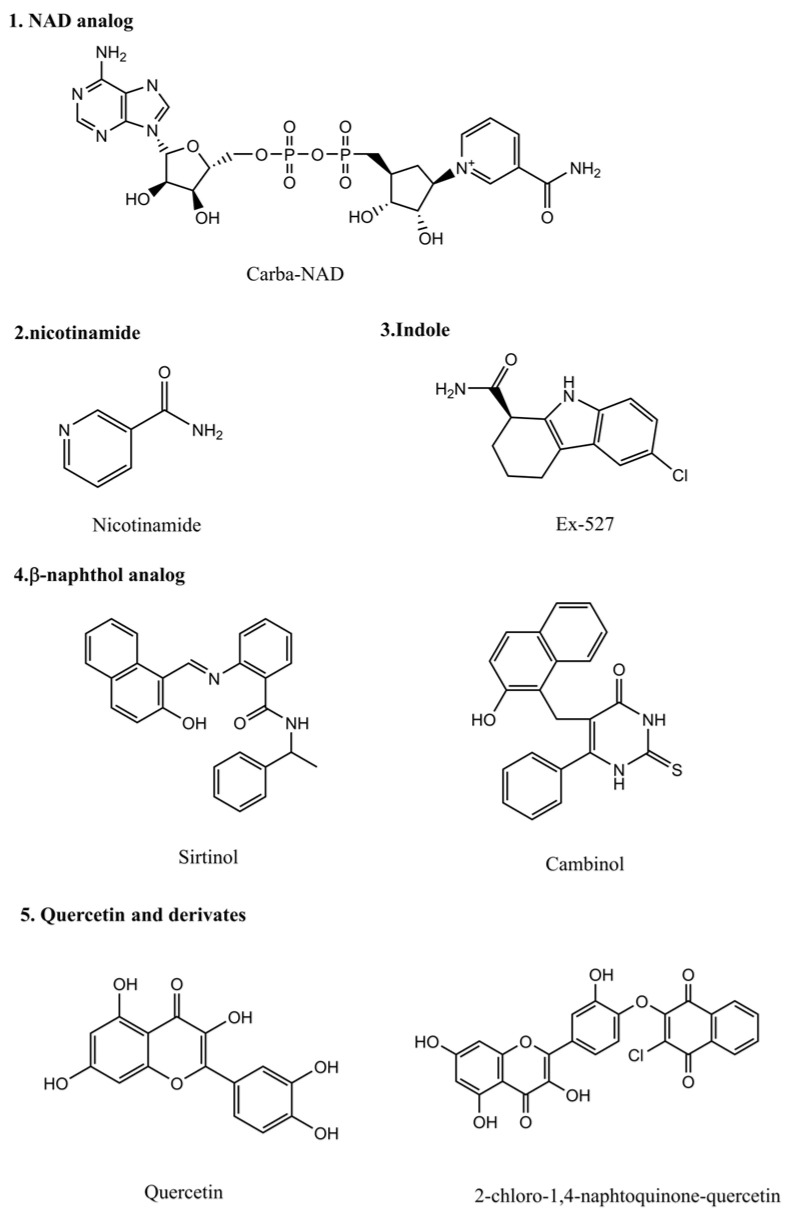
Chemical structure of SIRT inhibitors reported in the literature.

The first group of compounds, which inhibit the enzymatic activity of NAD-dependent deacetylase sirtuins, are non-hydrolyzable NAD analogs, such as carba-NAD [148] and nicotinamide (Figure 4) [149]. The latter, a byproduct of the deacetylase reaction catalyzed by sirtuins, is a non-selective sirtuin inhibitor and has the ability to inhibit nonmelanoma skin cancers. However, using SIRT6-coated magnetic beads [150], researchers identified quercetin and its derivatives as a group of SIRT6 inhibitors [151,152]. The indole compound, Ex-527 (Figure 4), was shown by high-throughput screening to be a selective SIRT1 inhibitor [153], and the β-Naphthol analogs are identified to be SIRT2 inhibitors (Figure 4) [154].

## 4. HDAC Inhibitors Promote Cisplatin Cytotoxicity

As HDAC inhibitors regulate both intrinsic biological and pathological processes via promoting histone acetylation and changing the chromatin structure, they have been reported to enhance the cytotoxicity of DNA-targeting anticancer drugs [14], in addition to their inherent anticancer activity [124,125,126]. Kim et al. showed that HDAC inhibitors TSA and SAHA (Figure 2) increased the cytotoxicity of a few anticancer drugs that target DNA or enzymes acting on DNA, e.g., etoposide (also named VP-16, a Topo II inhibitor), doxorubicin, and cisplatin [14]. Cisplatin is a well-recognized genotoxic drug. It exerts its anticancer function through forming crosslinking DNA adducts, in particular, 1,2-crosslinking DNA adducts, to distort DNA duplex structure [6,7,8]. It has been commonly accepted that HDACi enhance cisplatin anticancer activity via elevating histone acetylation. HDACi-induced increase in acetylation of histones neutralizes the positive charge of lysine residues in histones, weakening charge-dependent interactions between histones and nucleosomal DNA [155]. As a consequence, the elevated histone acetylation increases DNA accessibility by cisplatin, leading to the formation of more crosslinking Pt-DNA adducts and DNA lesions [14]. Further, the HDACi-induced increase in cisplatin lesions on DNA alters the action pathways of cisplatin, triggering and/or enhancing diverse biological processes and signaling pathways, such as apoptosis, cell cycle arrest, ROS level generation, and tumor suppressor transcription [14]. However, the HDAC 1/2 inhibitor valproic acid (VPA, Figure 2) was shown to increase GPX-4 expression and reduce lipid peroxidation, as a consequence inhibiting ferroptosis to protect the kidney against cisplatin-induced injury [156,157].

### 4.1. TSA

TSA is a fermentation product of Streptomyces. It was initially used as an antifungal agent [158]. TSA contains a hydroxamic acid moiety as its functional group, which confers this compound with potentials of HDAC inhibition and cancer therapy [159]. An early investigation revealed that pretreatment of four human cancer cell lines and two normal epithelial cell lines with TSA increased the cytotoxicity of several anticancer drugs, including cisplatin, in a cell-type-dependent manner [14,17]. Interestingly, the pretreatment with TSA promoted the cytotoxicity of cisplatin, but treating the cells in the reverse order had no effect on the activity of the anticancer drug. This means that it is TSA-induced alteration in the chromatin structure rather than TSA’s inherent anticancer activity that contributes to the promotion of cisplatin’s anticancer efficiency [14,17]. Our recent work demonstrated that TSA at a low concentration of 1 μM could promote cisplatin cytotoxicity towards human A549 non-small cell cancer cells by 3.4-fold, while TSA itself showed only a moderate cytotoxicity with an IC_50_ of 36 μM against the same cell line. We applied secondary ion mass spectrometry (SIMS) and inductively coupled plasma mass spectrometry (ICP-MS) to image/quantify the uptake of cisplatin in A549 cells co-treated with cisplatin and TSA and found that TSA increased Pt accumulation in A549 cells, leading to the formation of more cisplatin–DNA adducts [17]. These results strongly support that the TSA enhancement to cisplatin cytotoxicity is attributed to TSA-induced change in histone acetylation and chromatin structure, rather than to TSA inherent anticancer activity.

How TSA-induced histone hyperacetylation impacts the downstream biological process to promote cisplatin cytotoxicity against cancer cells is a crucial scientific issue attracting the attention of researchers in this field. A previous study reported that when A549 cells were treated with TSA and cisplatin, antiapoptotic factor cFLIP was shown to be downregulated, which activated caspase-8 to promote apoptosis [160]. For the cisplatin-resistant human bladder cancer cell line T24R2, concomitant TSA treatment increased the expression of cleaved caspase-3/9 and proapoptotic Bad and Bax, enhancing cisplatin-induced apoptosis [161]. Similarly, TSA upregulated p71 and induced Bax-dependent apoptosis to promote sensitivity of cisplatin towards cisplatin-resistant ovarian cancer cells [162]. Moreover, the TSA-induced overexpression of death-associated protein kinase, which in turn activated DAPK signaling, was demonstrated to be a way in which TSA sensitized cisplatin-resistant A549 cells to cisplatin [163]. In our recent work, we demonstrated that TSA promoted cisplatin-induced apoptosis of A549 cells through activating caspases 3 and 6, which was accompanied by the increase in cleaved poly-ADP-ribose polymerase (PARP) and the degradation of lamin A&C attributed to the presence of TSA [17]. Our proteomics analysis data also indicated that TSA co-treatment led to the upregulation of the transcription factor signal transducer and activator of transcription 1 (STAT1) and the hydrolase sterile alpha motif and histidine-aspartate domain-containing protein 1 (SAMHD1) via IFN-γ/α/β signaling, while it downregulated the intercellular adhesion molecule 1 (ICAM1) and CD44. These effects collectively contribute to enhancement in cisplatin efficiency and reduction in cell migration [17].

**Figure 5:**
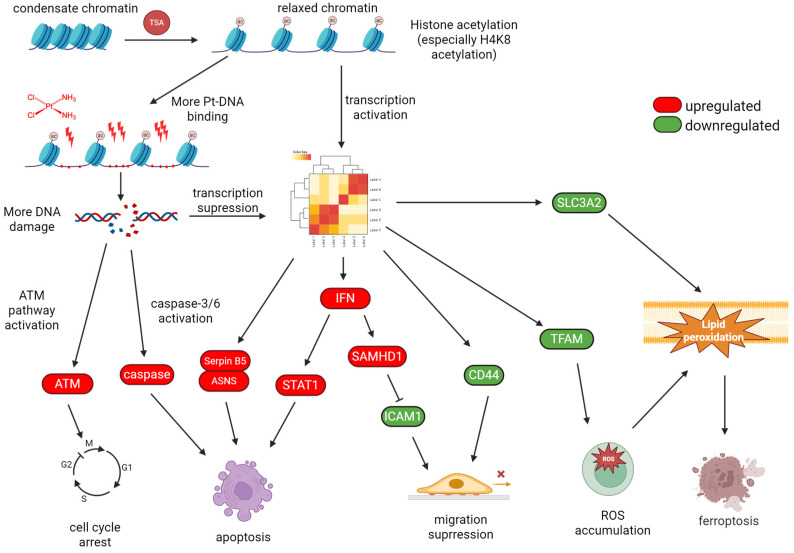
Proposed mechanisms by which TSA promotes cisplatin cytotoxicity. Adapted from our recent publication [17].

Cisplatin could cause G2/M arrest, and cells in the G2/M phase are most sensitive to cisplatin because some crucial events, such as sensing cisplatin-induced DNA damage and initiating the apoptotic process, are restricted to this phase [164,165]. Our results also showed that TSA enhanced cisplatin-caused G2/M arrest through activating the ATM-CHK2-Cdc25c-CDK1 pathway (Figure 5) [17]. The activation of this pathway is also attributed to the G2/M arrest of T24R2 cells caused by cisplatin in the presence of TSA [161].

However, a few studies unveiled that the TSA-induced increase in the formation of Pt-DNA adducts was not always the mere factor promoting cisplatin cytotoxicity. For instance, in the cisplatin-resistant human epidermoid cancer cell line KCP-4, TSA restored volume-sensitive Cl^−^ channel activity, which might increase the entrance of cisplatin and the efflux of Cl^−^. The reduction in Cl^−^ concentration facilitates the hydrolysis of cisplatin inside the cells, increasing cisplatin activation to promote the effectiveness of the drug [166]. Another report revealed that in the human A2780 ovarian cancer cells, TSA did not change the cisplatin accumulation and DNA damage inside cells; instead, it promoted cisplatin-induced apoptosis by activating caspase-9, autophagy by increasing LC3-II expression, and cell cycle arrest via elevating p21 expression [167]. Using mass spectrometry (MS)-based quantitative proteomics analysis, we found the solute carrier family 3 member 2 (SLC3A2) and the mitochondrial transcription factor A (TFAM) were downregulated when cells were exposed to cisplatin in the presence of TSA. SLC3A2 causes reduction in glutathione biosynthesis and inactivation of GPX4, while under-expression of TFAM induces ROS generation. These regulations synergistically promote ferroptosis via elevating lipid peroxidation, contributing to the promotion of cisplatin cytotoxicity by TSA (Figure 5) [17].

It is notable that TSA has also been reported to protect normal cells from cisplatin toxicity. Ma et al. showed that when normal kidney cells (HK-2 and mTEC cells) were co-treated with cisplatin and TSA, the TSA-induced upregulation of bone morphogenetic protein 7 (BMP-7) reduced the nephrotoxicity of cisplatin, protecting the kidney cells from cisplatin-induced acute kidney injury (AKI) [156]. Activating autophagy is also a crucial way for TSA to protect the kidney from injury caused by cisplatin [168,169]. Moreover, TSA was shown to upregulate the anti-inflammatory protein activated microglia/macrophage WAP domain protein (AMWAP) in epithelial cells, consequently suppressing renal epithelial cell apoptosis and reducing cisplatin nephrotoxicity [170]. In addition, TSA could also upregulate proline–serine–threonine phosphatase-interacting protein 2 (PSTPIP2) to rescue cisplatin-induced AKI [171].

Theoretically, acetylation can occur at any lysine residue in the flexible NH_2_-terminus of histones [16]. However, acetylation of which histone(s) and which lysine residue(s) plays crucial roles in promoting cisplatin cytotoxicity has remained unclear. Recently, we applied MS-based quantitative proteomics analysis to profile the acetylation of the DNA-packaging histones (H2A, H2B, H3, and H4) in A549 cells treated with cisplatin alone or in the presence of TSA so as to correlate the acetylation level of each lysine residue in histones and the activity of cisplatin. We revealed that the acetylation of H4K8 and H2BK20, in particular the former, showed a good positive correlation with the TSA-induced increase in cisplatin efficiency, as evidenced by the increase in level of the DNA-bound platinum and DNA double-strand break (DSB) marker γH2AX [17]. This finding provides a novel insight into the design and development of HDAC inhibitors, which could more effectively improve the clinical application of the first-line chemotherapeutic cisplatin.

### 4.2. SAHA

SAHA is a TSA analog, and the hydroxamic acid moiety is also its functional group (Figure 2) [172]. This structural similarity between SAHA and TSA confers SAHA with similar functions of impacting cisplatin efficiency. For instance, SAHA could increase cisplatin-induced apoptosis by activating caspases 4/12 [173]. Meanwhile, SAHA downregulated antiapoptotic factors, such as XIAP and Bcl-2, resulting in activation of caspase-3 to promote cisplatin-induced apoptosis [174]. Similar to TSA, SAHA-induced activation of autophagy was found to be a crucial way for SAHA to protect the kidney against injury caused by cisplatin [168,169].

Compared with TSA, however, SAHA not only increases the formation of cisplatin–DNA adducts but also changes the distribution of Pt-DNA adducts in the whole genome, enhancing the damage of some particular genes [175]. In HSC-3 cells, SAHA was shown to be able to reduce intracellular glutathione (GSH) level while it elevated cellular ROS level, which synergistically changed the intracellular redox status, enhancing cisplatin cytotoxicity [176,177]. Moreover, when PF49 and BHT-110 cells were treated with cisplatin and SAHA, PD-L1 expression was elevated, potentiating the antitumor action of cisplatin [178]. When H209 and H146 cells were cotreated with SAHA and cisplatin, the acetylation level of α-tubulin was promoted, leading to instability of the protein and more cell apoptosis [179]. When the oral squamous cell carcinoma cells were exposed to cisplatin and SAHA, eIF2α and protein phosphatase 1 (PP1) were activated. The former effect induced endoplasmic reticulum stress, while the latter decreased AKT phosphorylation, resulting in cell death [173]. In addition, research found that in mice, the combination of cisplatin and SAHA impacted the mouse cellular microenvironment and modified the internal tumor structure to reduce tumor growth [180].

### 4.3. VPA

Due to its safety for long-term use, valproic acid (VPA, Figure 2) was initially used as an antiepileptic drug, though it is a weak HDAC inhibitor compared with TSA and SAHA [181,182]. Afterwards, its own inherent anticancer activity and the potential to improve cisplatin effectiveness have also attracted many studies.

Rikiishi and co-workers reported that VPA sensitized the neuroblastoma UKF-NB-4 cell line to cisplatin through caspase-3-dependent induction of apoptosis, whereas this HDACi did not change the effectiveness of the anticancer drug vincristine, which acts through a mechanism different from that of cisplatin [183]. These results again verify that the synergistic anticancer effect of cisplatin combined with HDAC inhibitors results from the HDACi-induced changes in chromatin structure, but not from HDACi inherent anticancer activity. Surprisingly, somehow, for the UKF-NB-4 cells, the VPA-induced augment in cisplatin effectiveness was produced only when the cells were pre-treated with cisplatin prior to VPA exposure or when the cells were treated simultaneously with cisplatin and VPA. This contrasts with the results reported by Kim et al., who demonstrated that HDACi-mediated promotion of cisplatin cytotoxicity was produced only when the cancer cells were pre-treated with the HDAC inhibitors before the cells were exposed to anticancer drugs like cisplatin [14]. This difference may be attributed to differences in the types of cancer cells that the researchers used in their works. This also provides a rationale that the clinical efficiency of the combined use of cisplatin and HDAC inhibitors probably depends on the types of cancers treated.

Despite there being no similarity between the chemical structures of VPA and the hydroxamic acid derivatives TSA and SAHA, VPA has been shown to promote the cytotoxicity of cisplatin via similar ways to those of TSA and SAHA. For example, it has been reported that in cisplatin-resistant ovarian cancer cells, VPA increased DNA damage caused by cisplatin and ROS accumulation, promoting cisplatin efficiency [184]. When PF49 and BHT-110 cells were treated with the combination of cisplatin and VPA, PD-L1 expression was elevated, potentiating the antitumor action of cisplatin [178]. On the other hand, VPA has been reported to promote cisplatin cytotoxicity via different ways from those of TSA and SAHA. For instance, VPA was shown to increase cytotoxicity of cisplatin against bladder cancer via reducing survivin expression [185]. In cisplatin-resistant ovarian cancer cells, VPA promoted the expression of the tumor suppressor phosphatase and tensin homolog (PTEN) to resensitize cells to cisplatin [184]. The VPA–cisplatin combination was also shown to increase the expression of acyl-CoA synthetase long-chain family member 1 (ACSL1) while decreasing the expression of fatty acid synthase (FASN), which altered lipid metabolism, especially fatty acid oxidation and lipid synthesis, reducing cisplatin resistance [186].

It is notable that when normal kidney cells, HK-2 and mTEC cells, were co-treated with cisplatin and VPA, Bcl-2 was upregulated while BAX was downregulated, inactivating caspase-3 and reducing apoptosis of the kidney cells. These effects in turn resulted in attenuating acute kidney injury (AKI) induced by cisplatin [156]. It was shown that in the cisplatin-treated HK-2 cells, VPA also augmented GPX4 activity, eliminating ROS in cells and inhibiting ferroptosis to protect kidneys [157].

### 4.4. Others

A series of newly synthesized compounds with 2-aminoanilides that could inhibit HDAC1, HDAC2, and/or HDAC3, also lead to caspase-3/7 activation and induction of apoptosis in A2780, Cal72, and Cal27CisR cells, suggesting pan or class I/IIb HDAC inhibitors are not necessary to increase apoptosis induced by cisplatin [187].

Cisplatin combined with sodium butyrate increased the cell population in the G2/M phase [188]. Chidamide, an HDAC1/2/3/10 inhibitor, combined with cisplatin, carboplatin, or oxaliplatin, also elevated cell proportion in the G2/M phase [189]. Apigenin could inhibit HDAC activity and increase the expression of p21, and when cells were treated with cisplatin and apigenin, more cells were arrested at the G2/M phase than when treated with cisplatin alone [190].

Notably, unlike TSA and SAHA, HDAC inhibitor YWC1 increases DNA damage caused by cisplatin through impairing DNA repair [191]. Belinostat and romidepsin, combined with cisplatin, could effectively increase DNA damage in SCLC cells only when the cells were simultaneously treated with cisplatin and belinostat or romidepsin [192].

A newly designed HDAC inhibitor, S11, increased acetylation of H4 in the ornithine decarboxylase antizyme 1 (OAZ1) promoter region, resulting in upregulating cancer suppressor protein OAZ1 and resensitizing cisplatin-resistant A549 cells to cisplatin [193]. Furthermore, in HSC-3 cells, NaB and MS-275 (Figure 2) were shown to elevate cellular ROS to enhance cisplatin cytotoxicity [177].

It has been reported that ACY1215, an HDAC6 inhibitor, raised the acetylation of K129 in estrogen-related receptors α (ERRα), inducing ubiquitination and degradation of ERRα and restoring chemosensitivity of osteosarcoma cells to cisplatin [194]. EX527 (Figure 4), a SIRT1 inhibitor, increased the acetylation of K223 in forkhead box K2 (FOXK2), which could promote mitotic catastrophe, enhancing cisplatin chemosensitivity [195].

Tubastatin A (Figure 2) was demonstrated to improve SOD activity in cisplatin-treated kidneys, lowering oxidative stress and reducing kidney injury caused by cisplatin [167]. Quercetin (Figure 4), a SIRT inhibitor, could also increase SOD level and reduce ROS-mediated mitochondrial damage and inflammation through Nrf2/HO-1 and p38MAPK/NF-κB p65/IL-8 signaling pathways, mitigating cisplatin-induced apoptosis and antagonizing cisplatin-caused cardiotoxicity [196].

## 5. Perspectives and Remarks

### 5.1. Conjugation of HDAC Inhibitors with Pt(IV) Prodrugs for Multitargeting Chemotherapy

**Figure 6:**
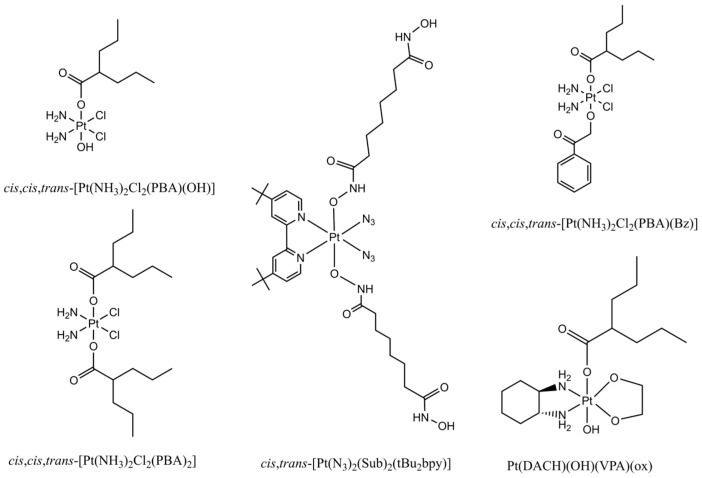
Chemical structure of a few synthetic Pt drugs containing HDAC-inhibiting units as axial ligands.

To overcome the tissue toxicity and resistance of cisplatin, Pt (IV) prodrugs have emerged as an attractive strategy. Unlike Pt(II), Pt(IV) has a six-coordination octahedral conformation and is chemically and pharmacologically more inert. However, when Pt(IV) complexes react with endo/exogenous reducing agents or are discomposed via light irradiation inside cancer cells, the inert Pt(IV) complexes can be reduced to Pt(II) species to exert anticancer activities. Meanwhile, the bioactive axial ligands coordinating to the Pt(IV) center are simultaneously released to interact with their biological targets, enhancing the effectiveness of the reduced Pt(II) species [197]. Given that HDAC inhibitors promote anticancer activity of cisplatin, the conjugation of the Pt(IV) center with HDAC inhibitors as one or two functionalized axial ligands (Figure 6) [198] has attracted increasing attention. For instance, Novohradsky et al. reported that when human ovarian cancer cells were treated with a Pt(IV) complex bearing two VPA ligands, *cis*, *cis*, *trans*-diamminedichlorido-bis(valproato)platinum(IV) [cisPt(VPA)_2_], the histone acetylation inside the cells was promoted more than when the cells were treated with free VPA, and DNA-bound Pt inside the cells was also markedly increased in comparison with that in the cells treated with cisplatin only [199]. This indicates that the conjugation of the Pt(IV) pharmacophore with the HDAC inhibitor (VPA) produces a synergetic effect, conferring the resulting complex, cisPt(VPA)_2_, higher cytotoxicity against the cancer cells than the combination of free cisplatin and VPA. It was reported that VPA, as one of the axial ligands in the Pt(IV) derivative of oxaliplatin, also elevated the accumulation of platinum in tumor cells, causing more nuclear DNA damage and showing higher cytotoxicity than oxaliplatin [200]. The HDAC inhibitor 4-phenylbutyrate (PBA) coordinates to the Pt(IV) derivative of cisplatin to form *cis*, *cis*, *trans*-[Pt(NH_3_)_2_Cl_2_(PBA)(Bz)] (Bz = benzoic), conferring the resulting complex a multitargeting feature. Inside cisplatin-sensitive A2780 or cisplatin-resistant A2780cis cancer cells, this Pt(IV) complex was reduced, inhibiting cell growth through increasing DNA damage and promoting ROS production [201]. Notably, a photoactivatable Pt(IV) complex *cis*, *trans*-[Pt(N_3_)_2_(Sub)_2_(tBu_2_bpy)] bearing two suberoyl-bis-hydroxamic acid (SubH) ligands was shown to have a synergetic anticancer activity after photoactivation, contributable to its dual functions, i.e., DNA targeting and histone deacetylase inhibition, in cancer cells [202]. Collectively, Pt(IV)–HDACi conjugation provides a new and promising strategy to develop novel Pt-based chemotherapeutic drugs.

### 5.2. HDACi Promote the Effectiveness of Other Chemotherapeutics and Radiotherapy

As mentioned earlier, besides cisplatin, the HDAC inhibitors, such as TSA, SAHA, and VPA (Figure 2), could also enhance the cytotoxicity of other anticancer drugs that target DNA or enzymes acting on DNA, e.g., doxorubicin and etoposide (also named VP-16) [14]. It was shown that VPA promoted doxorubicin-induced apoptosis through enhancing caspase-3 activity and elevating G2 arrest and that TSA increased the DNA double-strand break (DSB) caused by doxorubicin [203]. Additionally, TSA up-regulated PTEN expression to potentiate doxorubicin-induced apoptosis [204]. Pretreatment with TSA alone or TSA in combination with etoposide, a stabilizer for DSB, significantly sensitized HCC cells to apoptosis via inhibiting ERK phosphorylation, reactivating caspases and PARP, and inducing translocation of p53 and Bid to cytoplasm [205]. Two HDAC inhibitors, belinostat and romidepsin, were reported to effectively enhance DNA lesion induced by etoposide in SCLC cells only when either of the inhibitors and the drug were added simultaneously [192]. The combination of TSA with 5-fluorouracil (5-FU), paclitaxel (PTX), or irinotecan (SN38) also showed a synergistic anti-proliferative effect in OCUM-8 and MKN-74 cells through increasing the expression of p21, p53, DAPK-1, and the DAPK-2 genes [206]. Vorinostat (also known as SAHA, Figure 2) and LBH589 were reported to be able to downregulate thymidylate synthase (TS) gene expression in colon cancer cell lines, overcoming TS-mediated 5-FU resistance via enhancing cell cycle arrest and inhibiting cell growth [207]. The combination of a class I HDAC inhibitor, SNDX-275, with melphalan also showed a remarkable synergism on growth inhibition and DNA damage promotion [208], and TSA and VPA elevated the level of covalent DNA adducts generated by activated ellipticine in UKF-NB-3 and UKF-NB-4 cells [209]. The cotreatment of A549 cells with TSA/TXT or TSA/erlotinib was demonstrated to synergistically inhibit cell proliferation, induce apoptosis, and cause cell cycle delay at the G2/M transition. Moreover, treatment with TSA/TXT or TSA/erlotinib also leads to elevation of acetylation in alpha-tubulin and HSP90, decreasing expression of EGFR [210].

As chromatin structure could affect radiotherapy effect too, many HDAC inhibitors have been reported to be able to sensitize cancer cells to radiotherapy due to an increase in radiation-induced DNA damage [211]. For instance, NaB (Figure 2) increased radiation-induced DNA damage through suppressing expression of DNA DSB repair proteins Ku70 and Ku86 and DNA-dependent protein kinase catalytic subunit [212]. VPA led to radio-sensitization of LS174T and HTC116 cell lines through enhancing ionizing radiation (IR)-induced mitochondrial localizations of Bax and Bcl-xL, change in mitochondrial membrane potential, as well as cytochrome c release [213]. Furthermore, autophagy inhibition caused by TSA was shown to sensitize colon cancer cells to radiation [214]. Interestingly, HDAC inhibitors such as TSA, VPA, and phenylbutyrate were shown to be able to suppress cutaneous radiation syndrome, which limits the clinical application of radiotherapy, through promoting the healing of wounds caused by radiation and reducing later skin fibrosis and tumorigenesis. This clinical benefit was attributed to HDACi-induced suppression of the radiation-induced transforming growth factor β (TGF-β) and tumor necrosis factor α (TNF-α) [215].

Collectively, these results imply that HDAC inhibitors may provide a promising strategy for improving the effectiveness of cancer radiotherapy by inhibiting tumor growth while protecting healthy tissues.

### 5.3. HAT Inhibitor Also Enhances Cisplatin Anticancer Effect

Contrary to HDACs, histone acetyltransferases (HATs, also named lysine acetyltransferases (KATs)) increase histone acetylation levels to release genomic DNA to DNA-targeting drugs like cisplatin. Theoretically, therefore, overexpression or activation of HATs could promote the effectiveness of cisplatin. However, a number of studies have revealed that overexpression of HATs reduced anticancer activity or induced resistance of cancer cells against cisplatin [216,217,218].

Based on their structures and substrates, mammalian HATs could be divided into the following three major groups: GCN5-related N-acetyltransferases (GNAT) family, including HAT1, KAT2A (GCN5), KAT2B (PCAF), HAT4, and KAT9; p300 and CREB-binding proteins (p300/CBP); and the MYST family, including KAT5 (Tip60), KAT6A/B, KAT7, and KAT8 [219]. Some nuclear receptor coactivators, like SRC1, and transcription activators, such as TBP, TAF1, and TFIIIC90, also exhibit acetyltransferase activity [220]. It has been reported that HAT1 upregulated in hepatocellular carcinoma (HCC) facilitated HCC cell growth, and its knockdown could sensitize HCC cells to cisplatin-induced apoptosis [216]. Similarly, the overexpression of Tip60 was shown to be involved in cisplatin-resistant human epidermoid cancer cells and human prostate cancer cells, and the knockdown of Tip60 rendered cells sensitive to cisplatin [217]. Moreover, spermidinyl-CoA-based HAT inhibitors, which reduce histone acetylation, were reported to be able to arrest DNA synthesis and inhibit DNA damage repair, which consequently enhanced cellular sensitivity to cisplatin [218]. Kohno et al. reported that three HAT genes, Clock, Tip60, and PCAF, are overexpressed, endowing an antiapoptotic phenotype by promoting glutathione biosynthesis and the expression of E2F1 and DNA repair genes [221]. These studies collectively show that HAT inhibition could also promote cisplatin cytotoxicity by enhancing cisplatin-induced DNA damage and apoptosis via reducing histone acetylation. This discordance between the functions of HADC inhibitors and HAT inhibitors implicates the complexity of the regulation of the important epigenetic modification, i.e., histone acetylation, to the anticancer activity of genotoxic drugs such as cisplatin, suggesting that the balance of epigenetic modification status plays a crucial role in cancer therapy.

## 6. Concluding Remarks

Cisplatin is a widely used DNA-targeting anticancer drug, and the architecture of chromatin packaging genomic DNA is one of the determinant elements for the effectiveness of this drug. HDACs reduce histone acetylation levels, but HDAC inhibitors lead to histone hyperacetylation, increasing DNA accessibility by the drug. Theoretically, therefore, HDAC inhibitors could promote the cytotoxicity of cisplatin by increasing DNA damage caused by the drug. Indeed, a large number of studies, including ours, have revealed that HDAC inhibitors, such as TSA, SAHA, and VPA, significantly enhance the cytotoxicity of cisplatin against a variety of cancers in vitro and in vivo. Due to the complexity of this epigenetic modification itself and its regulation of downstream biological and physiological processes, the underlying mechanisms by which HDAC inhibitors improve the effectiveness of cisplatin are complicated, and research results are even controversial. Based on our recent research and by reviewing the advances in this research topic, we classify the mechanisms of HDACi promoting cisplatin into five major pathways: increasing DNA damage caused by cisplatin, promoting cisplatin-induced apoptosis and/or ferroptosis, elevating ROS levels inside cells, facilitating cell cycle arrest, and promoting tumor suppressor transcription. Nevertheless, HDACi-induced reduction in ROS level and in ferroptosis, and HDACi-induced increase in the expression of anti-inflammatory factors were thought to be the main way by which HDAC inhibitors alleviate the side effects, e.g., acute kidney injury, caused by cisplatin. It is worth pointing out that the HDACi-induced promotion of the effectiveness of cisplatin may depend on the types of cancers and the order of treating cancer cells with cisplatin and HDAC inhibitors. It is also notable that the conjugation of HDACi as one or two axial ligands in Pt(IV) prodrugs produces a greater synergistic anticancer effect than the combination of cisplatin and HDAC inhibitors. Given that HDAC inhibitors could also improve the efficiency of other genotoxic anticancer drugs and radiotherapy, the combination of HDAC inhibitors and genotoxic drugs or radiotherapy may open a wider avenue for cancer therapy.

## Figures and Tables

**Table 1:** The classification and functions of HDACs.

Name	Classification	Location	Main Functions	Cancer-Related Progress	Type of Cancer Cells	Refs.
HDAC1	Class I	Nucleus	cell cycle, proliferation and apoptosis regulation, and multidrug resistance	triggering proliferation, angiogenesis, invasion, migration, and apoptosis suppression	lung cancer and breast cancer	[64,65]
				Multidrug resistance	neuroblastoma	[49]
HDAC2	Class I	Nucleus	proliferation and apoptosis regulation	Cancer proliferation and progression	breast cancer	[66]
				Cancer progression and resistance	colorectal cancer	[67,68]
				Higher order chromatin changes and cellular DNA damage responses in chemotherapy	ovarian cancer	[69]
HDAC3	Class I	Nucleus	proliferation and apoptosis regulation	Proliferation and differentiation	colon cancer	[47]
				Growth and metastasis	ovarian cancer	[70]
				Chemosensitivity decrease	prostate cancer	[71]
HDAC4	class IIa	Cytoplasm and nucleus	angiogenesis and differentiation	Tumorigenesis, EMT, and angiogenesis	lung cancer	[50,72,73]
				Angiogenesis	acute T cell leukemia	[50]
HDAC5	class IIa	Cytoplasm and nucleus	differentiation	MEL cell differentiation	erythroleukemia	[53]
				Migration promotion	colon cancer	[74]
				Hormone therapy resistance	ER + breast cancer	[75]
HDAC6	class IIb	Cytoplasm	angiogenesis, EMT, and migration	Angiogenesis	renal cell carcinoma	[50]
				EMT and migration	lung cancer	[51]
				Migration	acute T cell leukemia	[52]
HDAC7	class IIa	Cytoplasm and nucleus	morphology, migration, and forming capillary tube-like structures	Tumorigenesis	lung cancer	[76]
				Proliferation, invasion, and migration	colorectal cancer	[77,78]
HDAC8	Class I	Nucleus	cell proliferation, cell cycle, and differentiation	Proliferation promotion and apoptosis inhibition	hepatocellular carcinoma	[79]
				Antiapoptosis	colon cancer	[80]
				Cell cycle control, differentiation, and apoptosis	neuroblastoma	[55]
				Invasion	breast cancer	[81]
HDAC9	class IIa	Cytoplasm and nucleus	cell proliferation, chemoresistance, invasion, and angiogenesis	Invasion and angiogenesis	triple-negative breast cancer	[57]
				Poor prognosis and immune cell infiltration inhibition	renal cell carcinoma	[58]
				Drug resistance, proliferation, and autophagic flux	gastric cancer	[56]
HDAC10	class IIb	Nucleus and cytoplasm	ferroptosis inhibition and DNA damage repair	Ferroptosis inhibition and DNA damage repair	lung cancer	[59,60,61]
				Chemoresistance: drug efflux promotion and DNA damage repair	neuroblastoma	[82]
HDAC11	class IV	Nucleus and cytoplasm	proliferation and apoptosis regulation, stemness enhancement, and resistance	Stemness and drug resistance	hepatocellular carcinoma and lung cancer	[63,83]
				Metastasis	colorectal cancer and breast cancer	[84,85]
SIRT1	class III	Nucleus	apoptosis attenuation, cancer formation and development, migration and invasion promotion (oncogene), DNA repair promotion, and antiapoptotic protein upregulation (tumor suppressor)	Apoptosis attenuation	lung and breast cancer	[86,87]
				Cancer formation and development	Breast cancer	[88,89]
				Migration and invasion	colorectal cancer	[90]
				DNA repair promotion	osteosarcoma and lung cancer	[91,92]
				Antiapoptotic protein upregulation	breast cancer	[93]
SIRT2	class III	Cytoplasm	regulate mitosis and genome integrity (tumor suppressor)	Regulating mitosis and genome integrity	hepatocellular carcinoma	[94]
				Cisplatin sensitivity	ovarian cancer	[95]
				Regulating metabolism and inhibiting metastases	colorectal cancer	[96]
SIRT3	class III	Mitochondrion	reduce superoxide levels and maintain genomic stability (tumor suppressor)	Tumor growth suppression by inhibiting ROS production	colon cancer	[97]
				Decreasing mitochondrial superoxide levels	mammary tumor	[98]
SIRT4	class III	Mitochondrion	glutamine metabolism block and DNA damage response (tumor suppressor)	Glutamine metabolism block and DNA damage response	lung cancer	[99]
				Suppressed proliferation, migration, and invasion	colorectal cancer	[100]
				self-renewal promotion	breast cancer	[101]
SIRT5	class III	Mitochondrion	cell growth and drug resistance	Cancer cell growth, transformation, and drug resistance	lung cancer	[102,103]
				Migration	hepatocellular carcinoma	[104]
				Suppressing DNA damage and cisplatin resistance	ovarian cancer	[105]
SIRT6	class III	Nucleus	gene expression and metabolic process regulation, telomeric chromatin modulation, DNA repair regulation, and inflammation attenuation (tumor suppressor)	p53 and p73 apoptotic signaling activation	cervical carcinoma and breast tumor	[106]
				Cell growth suppression	hepatocellular carcinoma	[107]
				Apoptosis induction	endometrial cancer	[108]
SIRT7	class III	Nucleus	transformed state of cancer cell stabilization, tumor aggressiveness, and poor prognosis	Oncogenic transformation, aggressive tumor phenotypes, and poor prognosis	fibrosarcoma	[109]
				Cancer progression promotion	lung cancer	[110]

## Data Availability

Not applicable.
